# Revisiting the Logan plot to account for non-negligible blood volume in brain tissue

**DOI:** 10.1186/s13550-017-0314-z

**Published:** 2017-08-18

**Authors:** Martin Schain, Patrik Fazio, Ladislav Mrzljak, Nahid Amini, Nabil Al-Tawil, Cheryl Fitzer-Attas, Juliana Bronzova, Bernhard Landwehrmeyer, Christina Sampaio, Christer Halldin, Andrea Varrone

**Affiliations:** 1 Department of Clinical Neuroscience, Center for Psychiatry Research, Karolinska Institutet and Stockholm County Council, Stockholm, Sweden; 2 CHDI Foundation/CHDI Management Inc., Princeton, USA; 3 Karolinska Trial Alliance, Karolinska University Hospital, M62, SE-141-86 Stockholm, Sweden

**Keywords:** PET, Kinetic modeling, Logan plot, [^18^F]MNI-659, Blood volume, PDE10A

## Abstract

**Background:**

Reference tissue-based quantification of brain PET data does not typically include correction for signal originating from blood vessels, which is known to result in biased outcome measures. The bias extent depends on the amount of radioactivity in the blood vessels. In this study, we seek to revisit the well-established Logan plot and derive alternative formulations that provide estimation of distribution volume ratios (DVRs) that are corrected for the signal originating from the vasculature.

**Results:**

New expressions for the Logan plot based on arterial input function and reference tissue were derived, which included explicit terms for whole blood radioactivity. The new methods were evaluated using PET data acquired using [^11^C]raclopride and [^18^F]MNI-659. The two-tissue compartment model (2TCM), with which signal originating from blood can be explicitly modeled, was used as a gold standard.

DVR values obtained for [^11^C]raclopride using the either blood-based or reference tissue-based Logan plot were systematically underestimated compared to 2TCM, and for [^18^F]MNI-659, a proportionality bias was observed, i.e., the bias varied across regions. The biases disappeared when optimal blood-signal correction was used for respective tracer, although for the case of [^18^F]MNI-659 a small but systematic overestimation of DVR was still observed.

**Conclusions:**

The new method appears to remove the bias introduced due to absence of correction for blood volume in regular graphical analysis and can be considered in clinical studies. Further studies are however required to derive a generic mapping between plasma and whole-blood radioactivity levels.

**Electronic supplementary material:**

The online version of this article (doi:10.1186/s13550-017-0314-z) contains supplementary material, which is available to authorized users.

## Background

The introduction of graphical analysis procedures for quantification of brain positron emission tomography (PET) data has made it possible to avoid many of the problems of regular compartmental modeling, such as the necessity of model identifiability and validation. In the derivation of graphical analysis approaches, including the Logan plot [1], no particular model configuration is required. More specifically, regardless of model configuration, the differential equations can be written on a linear form where the slope of the linear part of the curve corresponds to the *volume of distribution* (*V*_T_). In addition, only two free parameters need to be estimated by regression, which can be obtained from a one-step procedure rather than from iterative fitting approaches. Graphical analysis is therefore computationally cheap, making it suitable for generation of parametric images.

The signal used for kinetic modeling is typically obtained by defining regions of interest (ROIs), in which the radioactivity concentrations are measured over time. In addition to the radioligand in brain tissue, a fraction of the measured signal will originate from radioligand still in blood vessels. In full pharmacokinetic modeling, this blood contribution is typically included in the models, and the value for the fractional blood volume (*vB*) is either fixed (typically to 0.05) or estimated together with the other model parameters.

Explicit correction for signal originating from vascular tissue is not yet included in graphical analysis approaches. Thus, unless a correction has been performed beforehand, the *V*_T_ obtained from Logan plot reflects radioligand concentrations in both tissue and blood. Also, Logan plot relies on the assumption that the signal originating from the blood pool can be approximated by the metabolite corrected arterial input function (see the “Methods” section for details). Many radioligands display a time-dependent blood-to-plasma ratio, and thus, this assumption may lead to a systematic bias in *V*_T_. Biased outcome measures are problematic in clinical studies as it may obstruct findings of differences between clinical populations.

We here present a modification to the Logan plot that takes into account the contribution of the blood signal. In contrast to the original Logan plot, the *V*_T_ values that are obtained reflect radioligand binding only in tissue, and the equations are modified so that the approximation that equates the input function to the whole blood signal is removed. The new method is then extended to a modified Logan plot based on reference tissue (Logan ref) [2], with population-based correction for blood signal.

The new methods were evaluated using PET data acquired with the dopamine D2 radioligand [^11^C]raclopride and the phosphodiesterase (PDE) 10A radioligand [^18^F]MNI-659 [3]. [^11^C]raclopride was selected since it was used in the original publication presenting the Logan plot [1]. [^18^F]MNI-659 was selected for two reasons. First, our initial analysis of these data revealed that the cerebellar time-activity curve (TAC) could not be explained by the two-tissue compartment model (2TCM) without fitting of the fractional blood volume. Second, poor agreement between cerebellar *V*_T_ values estimated with 2TCM and those obtained with Logan plot was observed. We hypothesized that a potential reason for this disagreement was the underestimation of the signal originating from the blood vessels, which was observed to be high from parametric *V*_T _images of [^18^F]MNI-659.

In summary, the primary objectives of this study were (1) to modify the Logan plot in order to account for non-negligible signal from blood vessels, (2) to use data acquired with [^11^C]raclopride as a proof of concept for the proposed modification, and (3) to investigate whether the modified version of Logan plot provides improved quantification of data acquired with [^18^F]MNI-659 compared to the regular Logan plot.

## Methods

### Subjects

Twenty-one control subjects were included and confirmed healthy according to psychiatric and medical history, physical examination, laboratory testing, electrocardiography, and MR imaging. Written informed consent was obtained from each subject prior to initiation of the study. All procedures were approved by the Ethics committee of the Stockholm region and performed in accordance with the ethical principles that have their origin in the Declaration of Helsinki and that are consistent with ICH/Good Clinical Practice. The radiation safety committee of the Karolinska University Hospital, Stockholm, Sweden, also approved the examinations.

### PET and MR measurements

Six subjects (all males, age 27.8 ± 7.8 years) were examined using [^11^C]raclopride, and 15 subjects (10 M/5 F, age 46.3 ± 11.1) were examined using [^18^F]MNI-659. For each subject, a plaster helmet was made that fixated the subject’s head to the PET system in order to minimize head motion during the acquisition [4]. PET examinations were performed using the high-resolution research tomograph (HRRT, Siemens, Knoxville, TN, USA). A transmission scan was performed prior to radioligand injection using a rotating ^137^Cs source for attenuation correction. The radioligand was administered as a bolus injection. For [^11^C]raclopride, emission data was acquired for 63 min, corrected for decay and random and scattered events, and reconstructed into 4D image volumes using the Ordinary Poisson Ordered Subset Expectation Maximization Algorithm, including modeling of the system’s point spread function. The [^11^C]raclopride acquisitions were reconstructed into a total of 26 time frames. For [^18^F]MNI-659, emission data was acquired for 93 min, processed similarly as the [^11^C]raclopride dataset, and reconstructed into a total of 38 time frames. The injected activities and masses were 390 ± 71 MBq and 0.5 ± 0.4 μg for [^11^C]raclopride, and 209 ± 37 MBq and 1.2 ± 0.6 μ g for [^18^F]MNI-659.

Radioactivity levels in arterial blood were measured via an automatic blood sampling system for the first 10 min (ABSS, Alogg Technologies, Mariefred, Sweden), followed by manual arterial sampling at the midpoint of each time frame. Plasma samples were obtained after centrifugation of arterial blood and analyzed using high-performance liquid chromatography for metabolites. The parent fraction and the whole plasma curve were linearly interpolated for time points intermediate to sampling.

For subjects examined with [^11^C]raclopride, MR imaging was performed using a 1.5 Tesla GE signa system (GE medical systems, Milwaukee, WI, USA). For subjects examined with [^18^F]MNI-659, MR imaging was performed using a 3 Tesla GE-MR750 MR system.

### Image analysis

PET images were corrected for head motion using a frame-to-frame realignment procedure previously described [5]. For qualitative visual assessment of radioligand distribution, parametric images of [^18^F]MNI-659 *V*_T_ were generated using the wavelet aided parametric imaging (WAPI) approach [6], with parameter settings optimized for HRRT data [7].

MR images were reoriented such that the line defined by the anterior and posterior commissures was positioned parallel to the horizontal plane and the inter-hemispheric plane parallel to the sagittal plane. Afterwards, the MR images were coregistered to a summation PET image, using a six degrees of freedom, rigid body, affine transformation implemented in SPM5 (Wellcome Department of Cognitive Neurology, University College, London, UK).

FreeSurfer v5.0.0 [8, 9] was used for definition of ROIs for caudate, putamen, pallidum, accumbens, and cerebellar cortex. The segmentation provided by FreeSurfer was coregistered to PET space and projected onto the dynamic PET image to obtain regional time-activity curves (TACs).

### PET data quantification

#### Fractional blood volume of cerebellum for [^18^F]MNI-659

The operational equation for the 2TCM is

1 $$ {C}_m(t)=\left(1- vB\right)\left({C}_p(t)\otimes \left({\varphi}_1{e}^{-{\theta}_1t}+{\varphi}_2{e}^{-{\theta}_2t}\right)\right)+ vB\cdot {C}_b(t) $$

where *C*_*m*_ denotes the model curve that is fitted to the measured TAC, *C*_*p*_ is the metabolite corrected input function, *φ*_*i*_ and *θ*_*i*_ are model parameters to be fitted, *C*_*b*_ is the radioactivity measured in whole blood, and *vB* denotes the fraction of the ROI consisting of vascular tissue. It is common to estimate a global value for *vB* using whole brain TAC and subsequently fix *vB* to this estimate while fitting the regional TACs. Alternatively, *vB* can be fitted together with the model parameters for each ROI, allowing *vB* to vary across brain regions. The cerebellar [^18^F]MNI-659 TAC was fitted with both these procedures to determine which strategy provided a better fit. Similar comparison for the other ROIs or for the [^11^C]raclopride dataset is not shown here since the procedures provided similar results.

#### Logan plot using input function

Assuming a generic pharmacokinetic model, the original derivation by Logan et al. starts from

2 $$ \underset{0}{\overset{t}{\int }}A\left(\tau \right) d\tau =-{\mathbf{Un}}^{\mathbf{T}}{\mathbf{K}}^{-\mathbf{1}}\mathbf{Q}\underset{0}{\overset{t}{\int }}{C}_p\left(\tau \right) d\tau +{\mathbf{Un}}^{\mathbf{T}}{\mathbf{K}}^{-\mathbf{1}}\mathbf{A}, $$

where *A*(*t*) corresponds to the signal contribution from tissue in a given ROI, and **Un** ^T^, **K**, and **Q** are matrices containing model specific parameters. For the case of the 2TCM, *A*(*t*) *= C*_*ND*_(*t*) *+ C*_*S*_(*t*), where *C*_*ND*_ and *C*_*S*_ denote concentrations of non-displaceable and specifically bound tracer, respectively. Moreover, **Un** ^**T**^ = [1 1], **Q** = [*K*_*1*_ 0]^T^, **A** = [*C*_ND_ *C*_S_]^T^, and $$ {\boldsymbol{K}}^{-1}=\left[\begin{array}{cc}-\frac{1}{k_2}& -\frac{1}{k_2}\\ {}-\frac{k_3}{k_2{k}_4}& -\frac{k_2+{k}_3}{k_2{k}_4}\end{array}\right] $$.

Note that, in the above matrices, **A** is time dependent whereas the others are not. Solving the matrix Eq. (2) results in

3 $$ {\int}_0^tA\left(\tau \right) d\tau =\frac{K_1}{k_2}\left(1+\frac{k_3}{k_4}\right){\int}_0^t{C}_p\left(\tau \right) d\tau +M(t)={V}_T{\int}_0^t{C}_p\left(\tau \right) d\tau +M(t) $$

where *V*_T_ represents the distribution volume of the radioligand in tissue, and *M*(*t*) = **Un** ^T^ **K** ^−1^ **A**(*t*). The expression for *M* will not be of interest for the derivation, and is therefore not further discussed.

In the original implementation of the Logan plot, the TAC measured in an ROI over time *t, ROI(t),* is modeled as *ROI*(*t*) = *A*(*t*) + *V*_*p*_ ∙ *C*_*p*_(*t*), where *V*_*p*_ denotes the volume of plasma in tissue [1]. This model is limited, since it does not take into account that radioligand bound to blood cells and radioactive metabolites are present in the vascular tissue. Rather, it assumes that the signal coming from the blood vessels equates that of metabolite corrected arterial input function. We here instead use a model that is commonly applied for full kinetic modeling, i.e.,

4 $$ ROI(t)=\left(1- vB\right)\cdot A(t)+ vB\cdot {C}_b(t) $$

Integrating Eq. (4), and insertion of Eq. (3) results in

5 $$ {\int}_0^t ROI\left(\tau \right) d\tau =\left(1- vB\right)\cdot {V}_T{\int}_0^t{C}_p\left(\tau \right) d\tau + vB{\int}_0^t{C}_b\left(\tau \right) d\tau +\left(1- vB\right)\cdot M(t) $$

For the radioligands used in this study, plotting the integral of *C*_*p*_ versus the integral of *C*_*b*_ results in a linear function for *t* > t* (Fig. 2), where t*, after preliminary analysis of the data*,* was set as the same linearization time as that used to measure *V*_T_ and DVR (see the “Discussion” section). The concentration in whole blood can thus be mapped to the input function by

6 $$ {\int}_0^t{C}_b\left(\tau \right) d\tau =\alpha {\int}_0^t{C}_p\left(\tau \right) d\tau +\beta, $$

where *α* and *β* represents the slope and intercept of the linear part of the curve (i.e., t > t*). Insertion of Eq. (6) into (5) and dividing by the measured tracer concentration in the ROI results in

7 $$ \frac{\underset{0}{\overset{t}{\int }} ROI\left(\tau \right) d\tau}{ROI(t)}=\left\{\left(1- vB\right){V}_T+\alpha \cdot vB\right\}\frac{\underset{0}{\overset{t}{\int }}{C}_p\left(\tau \right) d\tau}{ROI(t)}+{M}^{\hbox{'}}. $$

Plotting ${\int}_0^t ROI\left(\tau \right) d\tau / ROI(t)$ versus ${\int}_0^t{C}_p\left(\tau \right) d\tau / ROI(t)$ results in a linear curve for t > t*, and an estimate of *V*_T_ in only tissue can be derived from the slope of the linear curve, as

8 $$ {V}_T=\frac{slope-\alpha \cdot vB}{1- vB}. $$

As seen in Eq. (8), for high-density regions, the slope of the Logan plot will be a dominating factor and thus a reasonable estimate for *V*_T_ (*vB* is typically in the order of 0.03–0.08, and *α* is in the order of 0.7 for [^11^C]raclopride and 2.5 for [^18^F]MNI-659). For low-density regions, however, the signal contribution from blood may be non-negligible. The two radioligands evaluated in this study displays a strong agreement between regional *V*_T_ values obtained with the standard 2TCM to those obtained with the traditional Logan plot (without any modifications) in the target regions, whereas a systematic discrepancy was observed for cerebellum. For this purpose, the correction term obtained in Eq. (8) was applied only to cerebellum, with values for the fractional blood volume in the reference region, *vB* ^ref^, obtained from fitting of the 2TCM (Eq. (1)).

To verify that Eq. (8) indeed performs a correction for blood volume, intermediate validation step was performed. Since, in this dataset, measurements of the whole blood curves were available, the TACs can be corrected on beforehand, by subtracting the whole blood curve multiplied by an estimate of *vB* from the TACs before applying the Logan plot. The resulting *V*_T_ estimates are thus presumably free from contribution of signal originating from blood. These values were compared to those obtained from Logan plot applied to uncorrected TACs, with and without the correction described in Eq. (8). However, since the effect is presumably limited to regions with low densities of the target, this intermediate analysis was only performed for the cerebellar TACs.

Finally, the regional distribution volume ratio (DVR = *V*_T_/*V*_ND_) was calculated, where *V*_T_ from cerebellum was used as an estimate of *V*_ND_. The agreement between DVR from Logan Plot, with and without inclusion of explicit correction for *vB* in cerebellum (i.e., Eq. 8), was compared to DVR obtained from 2TCM.

Note that, in the above reasoning, the input functions and whole blood curves are assumed to be known. In most cases when a reference region is available, these entities are not measured for practical reasons. Thus, until this point, the theory presented serves as a basis for performing correction for *vB* when blood measurements are not available, which is discussed below.

#### Logan plot using reference tissue

The expression in Eq. (7) is valid for any ROI. Assuming that a suitable reference region exists, whose *V*_T_ is adequately close to the global non-displaceable distribution volume *V*_ND_, and with fractional blood volume *vB* ^ref^, the following expression for *C*_*p*_ can be derived

9 $$ \underset{0}{\overset{t}{\int }}{C}_p\left(\tau \right) d\tau =\frac{1}{\left(1-{vB}^{\mathrm{ref}}\right)\cdot {V}_{ND}+\alpha \cdot {vB}^{\mathrm{ref}}}\underset{0}{\overset{t}{\int }}R\left(\tau \right) d\tau +{M}^{\hbox{'}\hbox{'}}, $$

where *R*(*t*)denotes the TAC for the reference region. Insertion of Eq. (9) into Eq. (5) provides an expression from which, when plotted in a similar way as Eq. (7) above, a truly unbiased expression of DVR as a function of the slope of the plot, *vB* ^ref^, *vB* (in the target ROI), *V*_ND_, and *α* is obtained. However, if the true *V*_T_ in the target region can be approximated by the slope of the traditional Logan plot (i.e., the signal from blood in the target ROI is negligible, corresponding to *V*_T_≈*slope* in Eq. (7)), insertion of Eq. (9) into (7) results in

10 $$ \frac{\underset{0}{\overset{t}{\int }} ROI\left(\tau \right) d\tau}{ROI(t)}\approx \frac{V_T}{\left(1-{vB}^{ref}\right){V}_{ND}+\alpha \cdot {vB}^{ref}}\frac{\underset{0}{\overset{t}{\int }}R\left(\tau \right) d\tau}{ROI(t)}+{M}^{\hbox{'}\hbox{'}\hbox{'}}. $$

This assumption may not be true for all radioligands, but for the radioligands in the current study, the signal contribution from blood in the target ROIs were found to be negligible. Finally, by plotting ${\int}_0^t ROI\left(\tau \right) d\tau / ROI(t)$ versus ${\int}_0^tR\left(\tau \right) d\tau / ROI(t)$ in Eq. (10), a DVR value that is compensated for a non-negligible signal contribution from blood in the reference region is obtained,

11 $$ DVR\approx slope\cdot \left(1+{vB}_p^{ref}\left(\frac{\alpha_p}{V_{ND_p}}-1\right)\right). $$

For consistency, the derivation and the results from not assuming that the blood signal is negligible in the target TACs are provided in Additional file 1. When the input function is not measured, individual estimates of *vB* ^ref^, *V*_ND_, and *α* are not available. Therefore, when investigating the performance of Logan plot based on reference tissue, population-based estimates of these parameters were used, which is indicated by the subscripts *p* in Eq. (11).

Lastly, since population-based values for *vB* ^ref^, *V*_ND_, and *α* were used, the biological connection between the parameter value and each individual measurement is lost. Therefore, an optimized procedure to estimate *α* was explored. More specifically, given that a gold standard *V*_ND_ obtained from 2TCM is available together with estimates for *vB* ^ref^, a value for *α* can be computed from Eq. (8) so that the correction term produces perfect agreement between *V*_ND_ obtained with 2TCM and Logan plot (*α* = [*slope* – *V*_ND_(1-*vB*)]/*vB*). The values for *α* obtained using this approach are referred to as *calculated α*, whereas those obtained from Eq. (6) are referred to as *measured α.* Similarly as before, both calculated and measured values for *α* were only employed in a population-based manner.

When evaluating the correction term shown in Eq. (11), DVR obtained from 2TCM was used as a gold standard, to which DVR obtained from Logan plot with and without correction was compared. For each subject, the population-based estimates for *vB* ^ref^_*p*_, *V*_ND*p*_, and *α*_*p*_ (measured or calculated) were calculated as the average values from all remaining subjects examined with the same radioligand in a “leave-one-out” fashion.

### Statistical analysis

To evaluate whether including fitting *vB* in the 2TCM improved the fit of the cerebellar [^18^F]MNI-659 model curve as compared to the 2TCM with *vB* estimated from the fitting of the whole brain, the sum of squared residuals, *χ* ^2^, and model selection criteria scores were calculated, and an *F* test was performed.

Linear regression analysis and Bland-Altman plots were used to evaluate the agreement between DVR obtained from Logan plot and 2TCM, before and after correction of signal from blood. In addition to the Bland-Altman plots, the reproducibility score (RC), defined by 1.96*SD of the difference between methods was calculated,

12 $$ RC=1.96\cdot \sqrt{\frac{1}{N}\sum_{i=1}^N{\left({DVR}_j^{\mathrm{Logan}}-{DVR}_i^{2\mathrm{TCM}}-\frac{1}{N}\sum_{j=1}^N{DVR}_j^{\mathrm{Logan}}-{DVR}_j^{2\mathrm{TCM}}\right)}^2} $$

where *N* is the number of ROIs. A low RC indicates that the bias is equal throughout the different regions (small vertical spread in the Bland-Altman plot). Also, the existence of a proportionality bias was evaluated by determining whether the regression line through the points in the Bland-Altman plot was different from 0. A proportionality bias indicates that the disagreement between two methods depends on the actual measurement and is not equal throughout the measured range.

## Results

### Fitting of 2TCM for [^18^F]MNI-659

**Fig. 1:**
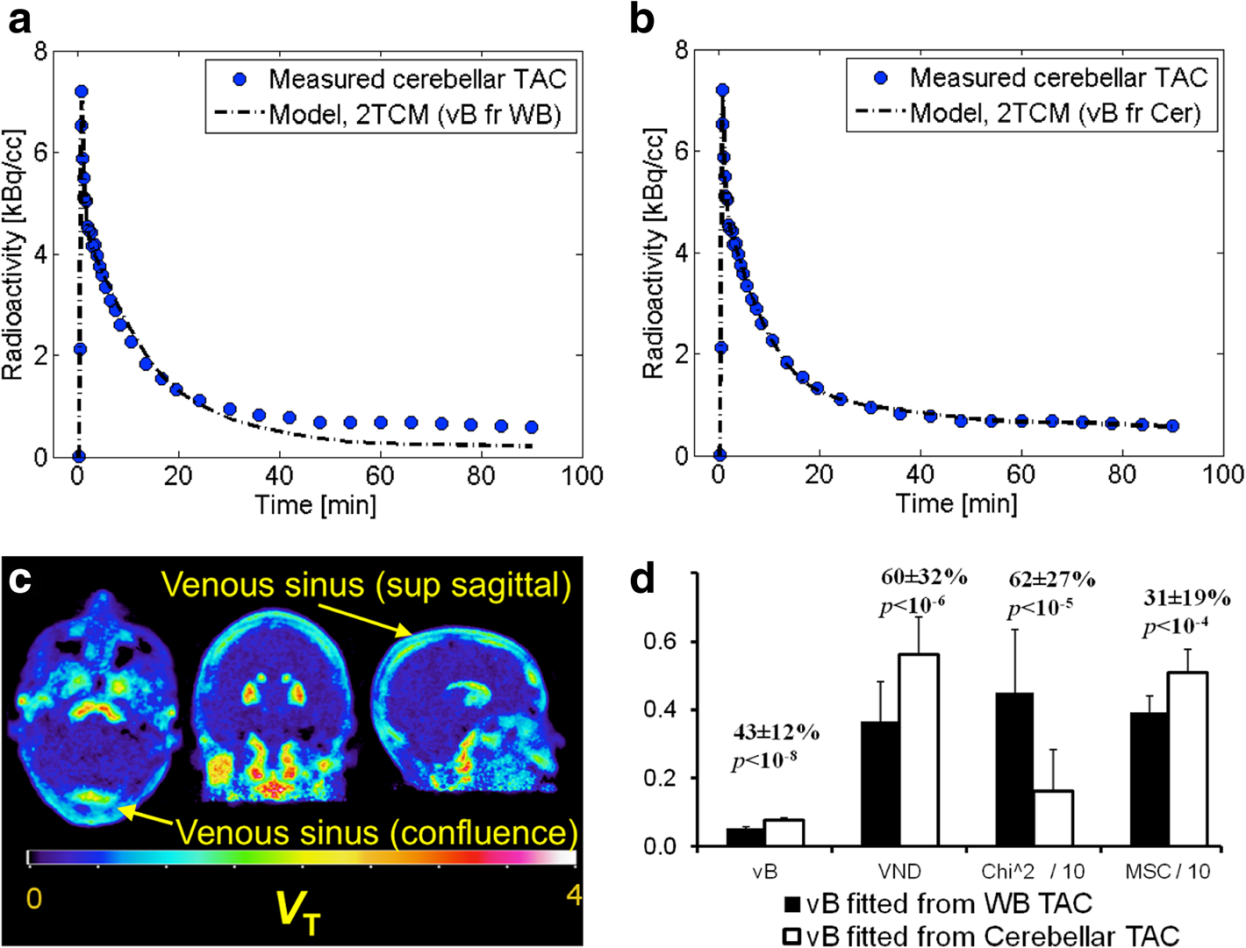
**a** Fit of the 2TCM to the [^18^F]MNI-659 cerebellar TAC using *vB* obtained from fitting the whole brain TAC, and **b** corresponding fit with *vB* fitted with the rate constants. The improved fit may be related to the high signal from blood vessels, seen in the parametric image shown in **c**. Fitting *vB* with the rate constants results in higher *vB*, higher *V*_ND_, lower *Χ* ^2^, and higher model selection criteria, shown in **d**. The numbers above the bars in **d** displays the percentage difference and the *p*-value from a paired t-test between the estimates obtained from the two procedures to fit *vB*

Figure 1 shows the effect of estimating *vB* together with the rate constants while fitting the cerebellar [^18^F]MNI-659 model curve as compared to using *vB* obtained from fitting the whole brain TAC. All goodness of fit measures, including the *F* test, indicated that estimating *vB* with the rate constants resulted in significantly improved fit (Fig. 1d, *p* < 10^−8^). Therefore, this procedure was selected as the gold standard method to estimate *V*_T_ in cerebellum (i.e., *V*_ND_). Interestingly, treating *vB* as a free parameter resulted in significantly higher values for *vB* and *V*_ND_ (Fig. 1d).

For [^11^C]raclopride, fitting *vB* together with the rate constants did not change model fit or the estimated values for *vB* and *V*_ND_ (not shown). For the sake of consistency with [^18^F]MNI-659, however, *vB* was fitted with the other rate constants.

### Logan plot using input function

**Fig. 2:**
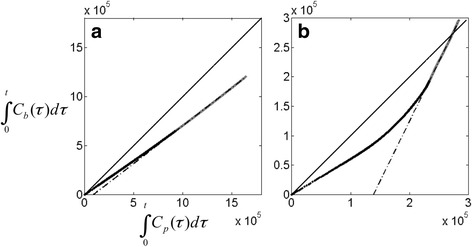
Integrals of whole blood radioactivity plotted against the integral of the metabolite corrected input functions for [^11^C]raclopride (**a**) and [^18^F]MNI-659 (**b**). For t > t*, the plot becomes linear (gray line), allowing for estimation of *α* in Eq. (6)

For every subject, plotting the integral of the whole blood radioactivity level versus the integral of the metabolite corrected input function according to Eq. (6) resulted in linear curves for *t* > t* (Fig. 2). The average (± SD) slopes (i.e., *α* in Eqs. (6) through (11)) are reported in Table 1. Although the actual slopes obtained were different for the two radioligands, similar coefficients of variation (SD/mean) across subjects were obtained (0.10 for [^11^C]raclopride and 0.14 for [^18^F]MNI-659).

**Table 1:** Average (SD) across all subjects of parameter estimates for Eq. 11

	[^11^C]raclopride	[^18^F]MNI-659
*vB* ^ref^ (2TCM)^*^	0.06 (0.03)	0.08 (0.01)
*V*_ND_ (2TCM)^*^	0.32 (0.03)	0.58 (0.13)
*α* measured	0.77 (0.08)	2.28 (0.32)
*α* calculated	0.69 (0.21)	1.51 (0.66)

*Values were estimated with the 2TCM. Note that, each subject’s measurement was omitted when calculating population-based estimates for that subject. Thus, the actual values used in Eq. 11 differed slightly from those reported in the table

**Fig. 3:**
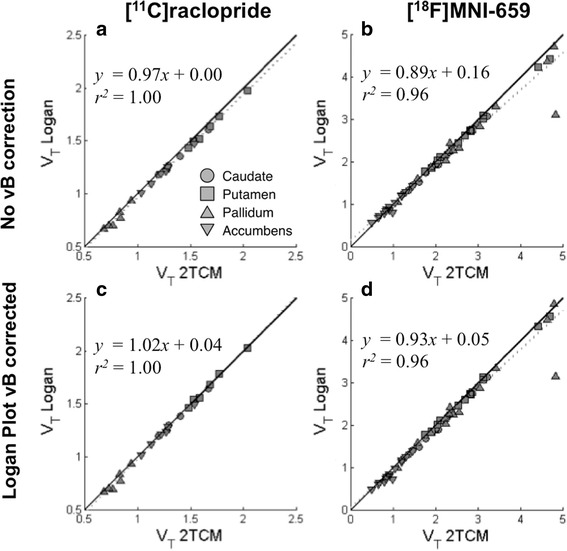
Distribution volumes (*V*_T_) obtained with 2TCM plotted against those obtained with Logan plot for all regions except cerebellum where *V*_T_ from Logan plot are (**a**, **b**) not corrected, and (**c**, **d**) corrected for signal from blood vessels, for [^11^C]raclopride data (**a**, **c**) and [^18^F]MNI-659 data (**b**, **d**)

For all regions except cerebellum, a good agreement between *V*_T_ obtained with 2TCM and *V*_T_ obtained using the Logan plot was observed, for both [^11^C]raclopride and [^18^F]MNI-659, illustrated by a regression line close to the identity line in Fig. 3. In addition, applying correction for *vB* according to Eq. (8) had little effect on the estimates of *V*_T_. These observations motivate that the correction shown in (9) was only applied to the reference region.

To verify that Eq. (8) indeed attempts to correct the Logan-derived *V*_T_ values for blood volume, the Logan plot was applied to cerebellar TACs that were already corrected for blood, in order to estimate *V*_ND_ free from blood contribution. When comparing *V*_ND_ obtained from Logan plot applied to uncorrected TACs to these estimates, a systematic overestimation was observed (15.7 ± 9.4% for [^11^C]raclopride and 20.0 ± 6.5% for [^18^F]MNI-659). Applying Eq. (8) with either measured or calculated *α* to compensate for this discrepancy substantially improved the agreement for [^11^C]raclopride (5.4 ± 3.6% and 7.8 ± 7.6% difference respectively). For [^18^F]MNI-659, meaningful improvement was only observed when calculated *α* was used (33.4 ± 25.3% with measured *α,* 4.1 ± 8.3% with calculated *α*). This suggests that, at least in part, the correction described in Eq. (8) compensates for blood contribution given that *α* is properly estimated.

**Fig. 4:**
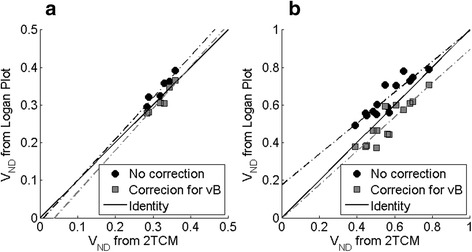
Distribution volumes (*V*_T_) obtained with 2TCM plotted against those obtained with Logan plot for cerebellum with and without the correction described in Eq. 8, for **a** [^11^C]raclopride and **b** [^18^F]MNI-659

Further, although Logan plot and the 2TCM provided similar estimates of *V*_T_ for all target regions, a systematic difference in cerebellum was observed (Fig. 4). The average absolute (± SD) difference between *V*_ND_ estimated with 2TCM and Logan plot was 6.5 ± 3.3% and 12.2 ± 8.2% for [^11^C]raclopride and [^18^F]MNI-659, respectively. Correcting the cerebellar *V*_T_ obtained from Logan plot for blood volume using Eq. (7) provided substantially improved agreement to 2TCM for [^11^C]raclopride (3.2 ± 2.6% absolute difference, and regression line close to the identity (Fig. 4)). For [^18^F]MNI-659, this procedure resulted in “over correction,” i.e., most subject appeared below the identity line in Fig. 4b, and 14.3 ± 9.3% absolute difference.

### Logan plot using reference region

Averages and standard deviations for the parameters used in Eq. (11) for both [^11^C]raclopride and [^18^F]MNI-659 are reported in Table 1. Note that, when applying Eq. (11), only population-based estimates of these parameters were used. Each subject’s measurement was omitted when calculating population-based estimates for that subject, resulting in that for each subject, the actual values used in Eq. 11 differed slightly from those reported in the table.

#### [^11^C]raclopride

**Fig. 5:**
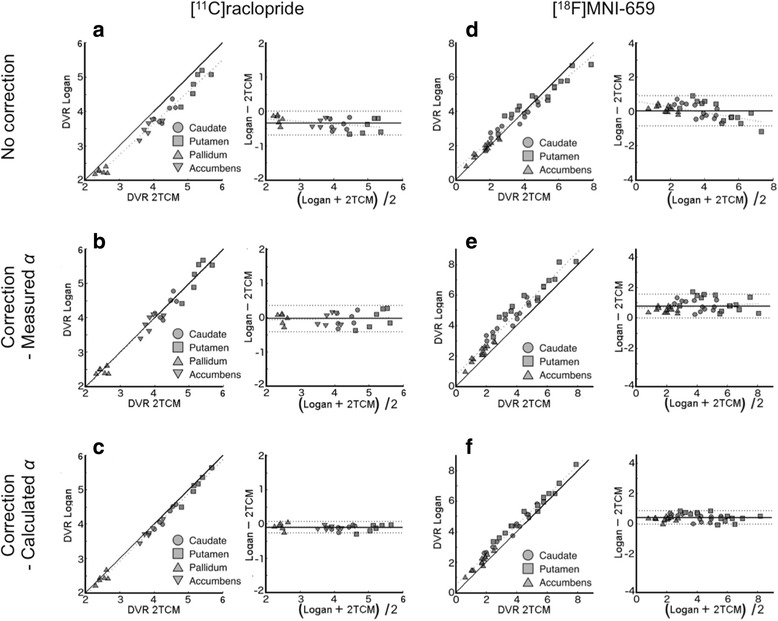
Distribution volumes ratios (DVR) obtained with 2TCM plotted against those obtained with Logan ref, and corresponding Bland-Altman plots. DVRs from Logan ref. are: not corrected (**a**, **d**), corrected using measured values for *α* (**b**, **e**), and calculated values for *α* (**c**, **f**). Left column (**a**, **b**, **c**) shows [^11^C]raclopride data, and right column (**d**, **e**, **f**) shows [^18^F]MNI-659 data

Regional DVR values obtained from Logan ref were systematically lower than those obtained from 2TCM (8.7 ± 4.0%, *p* < 10^−8^, Fig. 5a). The difference between the methods were however rather consistent across ROIs (slope in Bland-Altman plot was only different from 0 on a trend level (*p* = 0.05), and the spread was limited (RC = 0.35). This suggests that Logan ref does not introduce a considerable proportionality bias for [^11^C]raclopride (i.e., the methods do agree equally through the range of the measurements).

Correcting [^11^C]raclopride DVR obtained from Logan ref according to Eq. (11), using population-based values for *V*_ND_, vB^ref^, and *α* (measured) increased the agreement to DVR obtained from 2TCM (0.8 ± 5.0% difference, *p* = 0.46), Fig. 5b). Again, no proportionality bias was observed (slope in Bland-Altman plot not different from 0 (*p* = 0.63, RC = 0.39)). Lastly, applying the correction in Eq. (10) to DVR obtained from Logan ref, but using *α* values calculated from Eq. (8), with population-based values for *V*_ND_ and *vB* ^ref^ also improved the agreement to DVR values obtained from 2TCM (Fig. 5c, 2.5 ± 2.4% difference, *p* < 10^−5^). Similarly as before, no proportionality bias was observed (*p* value for slope in Bland-Altman plot = 0.55), and the spread was reduced (RC =0.17).

#### [^18^F]MNI-659

The results for correcting DVR obtained for [^18^F]MNI-659 for contribution from blood is compiled in Fig. 5d–f and in Table 2. In general, the results obtained for pallidum differed from the other ROIs in terms of agreement between 2TCM and Logan plot. As this analysis was to a large extent performed across ROIs in order to evaluate performance of the new method, results in Table 2 are presented both with and without pallidum included in the ROI set. Figure 5 however only includes the result with pallidum omitted from the analysis. The corresponding figure including pallidum is presented in the Additional file 1.

**Table 2:** Results from applying the correction described in Eq. (11) to DVR values obtained from Logan ref. and [^18^F]MNI-659. When the slope of the Bland-Altman plot ≠0, no average difference can be calculated due to proportionality bias

		Linear regression			Bland-Altman		% Diffference	
		Slope	Intercept	*r* ^2^	Slope	RC	Avg (SD)	*t* test (*p*)
With pallidum	No correction	0.74	0.81	0.93	*p* < 1e − 8	1.3	–	–
	Measured *α*	0.91	1.00	0.93	*p* = 0.10	1.1	−24.7 (20.4)	< 1e − 11
	Calculated *α*	0.89	0.71	0.95	*p* < 0.01	0.9	–	–
Without pallidum	No correction	0.83	0.59	0.96	*p* < 1e − 4	0.87	–	–
	Measured *α*	1.01	0.73	0.95	*p* = 0.32	0.79	−24.8 (20.4)	< 1e − 11
	Calculated *α*	0.98	0.47	0.98	*p* = 0.61	0.46	−13.4 (15.5)	< 1e − 4

Regardless of whether pallidum was included or not, DVR obtained from Logan ref displayed a systematic difference and a proportional bias compared to DVR obtained from 2TCM, as revealed by non-zero slope in the Bland-Altman plot. Due to this proportional bias, the percent difference between DVR obtained with 2TCM and Logan ref is presented for each ROI separately (caudate −4.1 ± 14.7%, *p* = 0.91; putamen −1.8 ± 14.0%, *p* = 0.60; pallidum 8.3 ± 16.3%, *p* < 0.05; accumbens −11.6 ± 16.8, *p* < 0.05). With pallidum included in the analysis, linear analysis revealed slopes < 1 and intercepts > 0 regardless of which correction method was applied (Table 2). When Eq. (11) was applied to DVR obtained from Logan ref, with measured values for *α*, the proportionality bias was removed (*p* = 0.10), but a considerable spread was still observed (RC = 1.1, and the standard deviation of the average difference between the methods was high (Table 2)). When calculated values for *α* were used, the proportionality bias was reintroduced, and the percent difference between methods was generally non-negligible (caudate −15.3 ± 11.6%, *p* < 10^−5^; putamen −12.6.0 ± 10.9%, *p* < 10^−4^; pallidum −1.7 ± 15.9%, *p* = 0.37; accumbens −23.9 ± 15.6, *p* < 10^−6^).

With pallidum excluded from the statistical analysis, the results were in general more easily interpretable. First, without any correction applied to DVR obtained from Logan ref, the slope in the linear regression analysis was < 1, and a non-zero slope in the Bland-Altman plot was observed (Fig. 5d, Table 2). Correcting the DVR values from Logan ref using Eq. (11) with measured values for *α* resulted in a slope close to 1 in the regression analysis and no proportionality bias (Fig. 5e). With this procedure, the percent difference between the methods was still ~ 25% (*p* < 10^−11^), and a considerable spread in the Bland-Altman plot was observed (RC ~ 0.8). Lastly, correcting DVR values from Logan ref using *α* values calculated from Eq. (8) with population-based estimates of *vB* ^ref^, *V*_ND_, and *α* resulted in a slope ~ 1 in the linear regression analysis, no proportionality bias (*p* ~ 0.6), reduced spread in the Bland-Altman plot (RC ~ 0.5), and reduced percent difference between DVR obtained with the different methods as compared to other correction methods (Fig. 5f).

## Discussion

Correcting for signal originating from blood vessels is typically not considered when performing kinetic modeling based on reference tissue. There is no theoretical rationale for not correcting for the blood vessels signal as it has been shown that, even if the target and the reference region are equally vascularized, the estimates of *BP*_ND_ will be biased [10]. Conversely, this bias can be understood intuitively as a *consequence* of different brain regions being equally vascularized; if *vB* is similar across brain regions, the actual contribution of signal from blood to each TAC will be equal. Thus, signal measured in regions associated with low density of binding sites will consist of a larger fraction of signal from blood than that measured from a region with a high density of binding sites. If no correction for the blood signal is performed, the signal measured from region with low density of binding sites is therefore more overestimated than signals measured from high-density regions. Following this reasoning, the overestimation will be largest in a region totally devoid of target receptors. If such region is used as a reference region, the error caused by the signal from blood vessels will propagate to every other region, resulting in systematic underestimation of *BP*_ND_ estimated from reference tissue approaches.

Although there is no theoretical rationale for not correcting for blood volume in reference tissue modeling, there is indeed a pragmatic one. Traditional correction for blood signal relies on the measurement of the radioligand concentration in arterial blood (i.e., *C*_*b*_ in Eq 1), whereas reference tissue models were originally designed to obviate the need of blood sampling during the PET measurement. Thus, if blood sampling is omitted, some bias in estimates of *BP*_ND_ is accepted. To our knowledge, a method to correct for the blood volume without performing arterial blood sampling has not been reported yet.

In this study, we have revisited the well-established Logan plot and derived a procedure that corrects either the distribution volume or the distribution volume ratio for signal originating from blood vessels, and we evaluated the procedure on PET data acquired using two radioligands. In agreement to the reasoning above, we found that for brain structures associated with high binding, the signal contribution from radioligand in blood vessels is negligible. For low-density structures however, particular consideration should be given to contribution from blood, as Logan Plot systematically overestimated *V*_ND_ as compared to 2TCM, which theoretically would lead to underestimation of *BP*_ND_ obtained from reference region approaches.

In this study, DVR obtained for Logan ref was indeed systematically underestimated for [^11^C]raclopride, whereas for [^18^F]MNI-659, the underestimation was observed only in the regions with the highest density of PDE10A. Underestimation of *BP*_ND_ obtained from Logan ref has repeatedly been reported, for instance for [^11^C]doxepin for the histamine H1 receptor [11], [^11^C]carfentanil for the μ-opioid receptor [12], [^11^C]ITMM and [^11^C]ITDM for the metabotropic glutamate receptor [13, 14], [^18^F]NMB for dopaminergic D2-like receptors [15], [^11^C]cimbi36 for the serotonin 2A and 2C receptors [16], [^11^C]flumazenil for GABA_A_ receptor [17], [^18^F]AZD4694 for amyloid *β* [18], [^11^C]AZ10419369 for serotonin 1B receptor [19], and [^11^C]PE2I for the dopamine transporter [20]. All these studies report that DVR (or *BP*_ND_) obtained with Logan ref was ~ 10–30% lower than that obtained with compartmental modeling, which is of the same order as the underestimation reported here. It is possible that these underestimations are an effect of not accounting for the blood signal.

To date, few attempts have been made to estimate the error introduced by a non-negligible blood volume in reference tissue modeling approaches. Since no real blood measurements can be assumed to be available, corrections for blood signal requires mapping the radioligand concentration in whole blood to the arterial input function, and then perform the necessary substitutions in the analytic expressions (similar to Eq. (5) in this study). A difficulty in this mapping is that the relationship between the whole blood concentration and the input function changes over time, and thus likely needs to be established for every radioligand separately. For the simplified reference tissue model (SRTM) and [^11^C]raclopride, Gunn et al. successfully predicted the bias caused by blood signal by inserting this relationship as a scalar value, approximated by plasma-to-blood ratio multiplied by the parent fraction at the end of the acquisition [21]. Using the same methodology however, Salinas et al. [10] observed only moderate agreement between predicted and estimated bias using simulated data obtained with input functions and blood-to-plasma ratio from [^11^C]PHNO. In the current study, a linear relationship between the integrals of the input function and the whole blood concentration was used (i.e., the slope in Fig. 2). The biological interpretation of this slope can be seen from the derivative with respect to time of Eq. (6); *α = C*_*b*_(*t*)*/C*_*p*_(*t*). Thus, a constant value for *α* indicates that, for t > t*, an equilibrium has occurred between the whole blood concentration and the input function (i.e., similar to what was assumed previously) [10, 21]. Since this procedure only provided good agreement for [^11^C]raclopride, more work may be needed to derive a generic theoretical mapping between *C*_*p*_ and *C*_*b*_. For instance, it is possible that the time for linearization (t*) between *C*_*p*_ and *C*_*b*_ should not equate that between plasma and tissue, as these are in essence independent processes. We here used equal t* based on inspection of the curves, and we acknowledge that such procedure is both radioligand specific and not well founded in theory. Until a generic theoretical mapping between *C*_*p*_ and *C*_*b*_ has been established however, we here provide a pragmatic solution: On a training dataset for which input functions are available, identify a value for *α* so that perfect agreement between *V*_ND_ estimated with Logan plot and 2TCM is obtained.

For [^11^C]raclopride, both these approaches resulted in close agreement between DVR from Logan ref and 2TCM. For [^18^F]MNI-659, however, meaningful improvements were only observed when *α* was calculated from Eq. (8). A possible explanation is that, of the parameters used in Eq. (11), *vB* ^ref^ and *V*_ND_^ref^ are reasonably easy to estimate, whereas *α* is associated with some uncertainty. Possibly, [^18^F]MNI-659 displays a faster metabolism and more complicated blood-to-plasma behavior, so that the integrals of plasma and whole blood do not display a perfectly linear relationship, even after an arbitrary late t*. In addition, from investigation of parametric images of distribution volume, it was discovered that the signal originating from blood is surprisingly high, indicating that the radioligand may accumulate in blood cells or vessel walls, which has been assumed for other radioligands [22]. Accumulation of [^18^F]MNI-659 binding in vascular tissue has some support from the literature. For instance, PDE10A expression appears to play an important role in pulmonary hypertension [23] and is expressed in the vasculature within skeletal muscles [24]. However, in vitro studies are needed to further verify the existence of specific binding of [^18^F]MNI-659 to vessel walls.

In this study, the expression used for Logan ref is different than that originally proposed by Logan et al., as the ratio of *C*_*T*_(*t*) and the population average of *k*_*2*_’ is not included in the independent variable. To justify this simplification, we calculated DVR using both approaches and found them to be in very close agreement (slope = 1.00, intercept = 0.00, *r* ^2^ = 1.00, details not shown).

Lastly, with regards to the newly developed radioligand [^18^F]MNI-659, the 2TCM could not properly describe the [^18^F]MNI-659 cerebellar time activity curve if fractional blood volume (*vB*) was fixed to the value found for the whole brain. Instead, the model consistently underestimated the TAC in the late part of the measurement. When *vB* was fitted individually for cerebellum, the model fit was significantly improved. In addition, the actual values for *vB* and *V*_ND_ obtained were significantly larger than those obtained when *vB* was fixed. This behavior is to some extent atypical for a PET radioligand, as the application of 2TCM is often not sensitive to the method used for estimation of *vB* (for instance, the same was not observed for [^11^C]racloride). The most probable explanation is that [^18^F]MNI-659 experiences a low extraction fraction in combination with a fast washout from tissue. This combination of properties results in that, at late times, a relatively large amount of radioligand is still in blood, and as the signal from tissue has washed out, the signal measured in the whole ROI consists to a large extent of the signal from blood vessels, and hence, the importance of estimating a correct fraction of vascular signal is emphasized.

## Conclusions

When applying 2TCM to [^18^F]MNI-659 PET data, the fractional blood volume in cerebellum should be fitted with the other model parameters. Moreover, if no correction for blood volume is applied, DVR values obtained from Logan ref may be underestimated. Using the methodology proposed in this study may compensate for this underestimation, without requiring blood sampling.

## Additional file

Additional file 1: Supplementary material. (DOCX 503 kb)
