# Supplementary material for: Revisiting the Logan plot to account for non-negligible blood volume in brain tissue
Source: EJNMMI Res. 2017 Aug 18;7:66. doi: 10.1186/s13550-017-0314-z (PMC5561763; doi:10.1186/s13550-017-0314-z)
Supplement: Supplementary file 1 — Supplementary material. (DOCX 503 kb) [file 13550_2017_314_MOESM1_ESM.docx]

Revisiting Logan plot to account for non-negligible blood volume.

Additional file 1

**S1. Derivation of correction for blood volume in both target region and in reference region.**

From equation (9), the following relationship between the parent radioligand concentration in arterial plasma (*C_p_(t)*) and the time-activity curve of the reference region, *R(t)*,

_
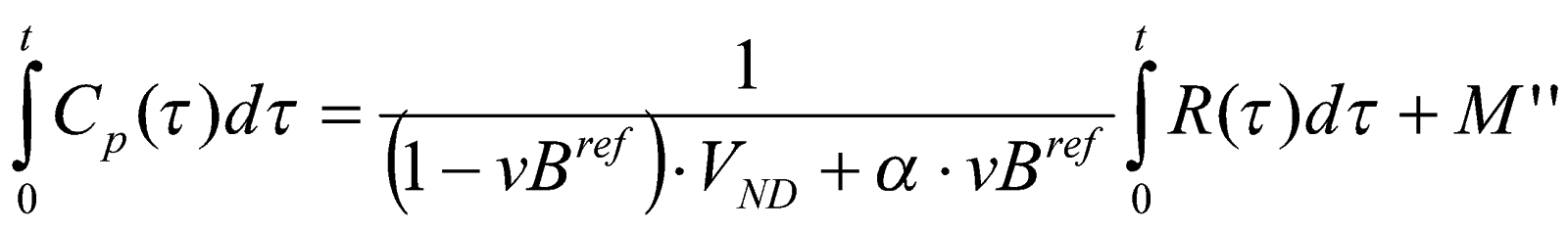
_. (S1)

Inserting this expression into equation (7) results in

_
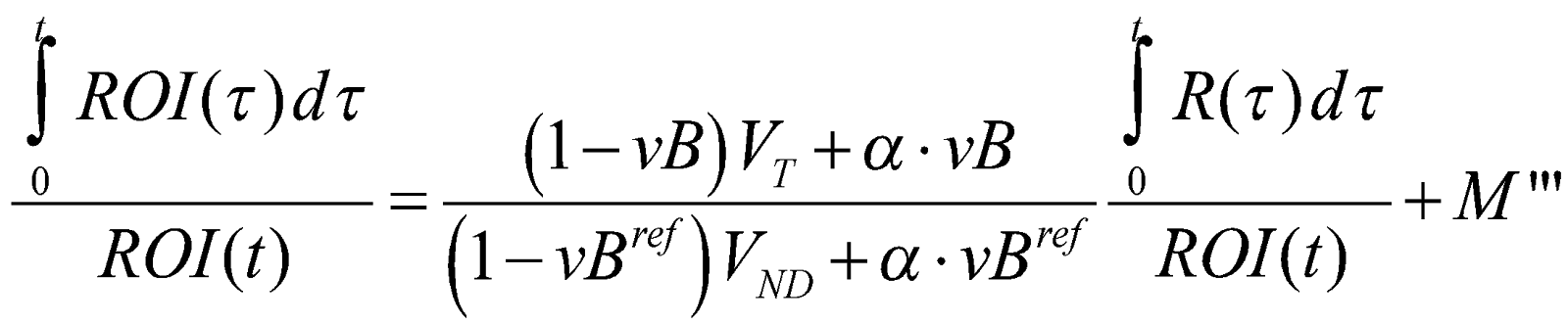
_, (S2)

where *vB* corresponds to the blood volume of the target region. The slope of the linear part of the plot $\frac{\int_{0}^{t} R(\tau)d\tau}{ROI(t)}$  versus  $\frac{\int_{0}^{t} ROI(\tau)d\tau}{ROI(t)}$ thus corresponds to

_
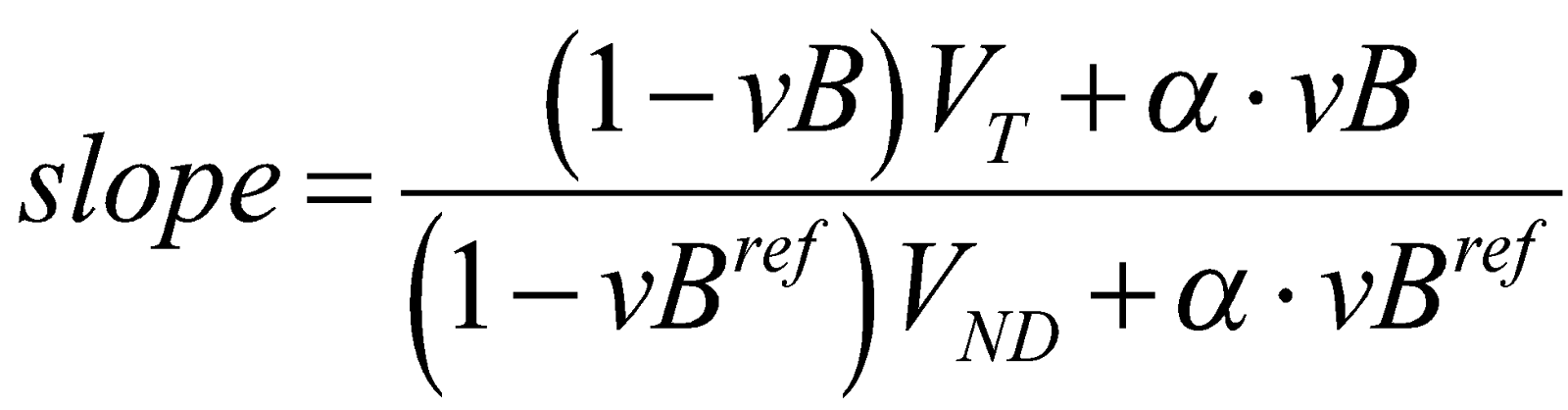
_. (S3)

Rearrangement of equation (S3) results in

_
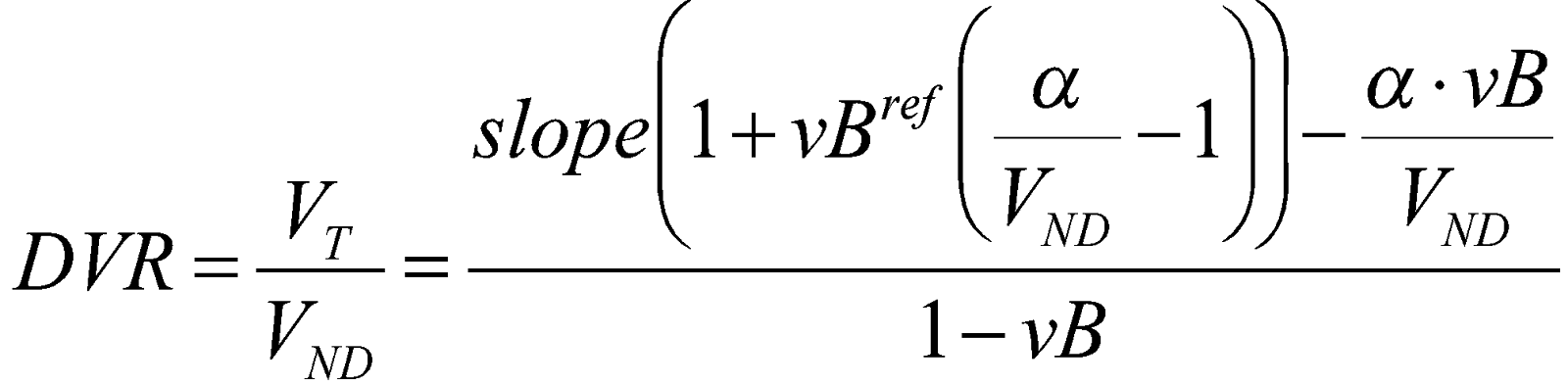
_ . (S4)

By using population based estimates *V*_ND_, *vB*, *vB^ref^*, and *α*, the DVR with no contribution from blood in both the target region and the reference region can be calculated (assuming the signal from blood in the target to be negligible corresponds to setting *vB* =0 in equation S4). The agreement to DVR obtained from 2TCM is shown in Figure S1 below.


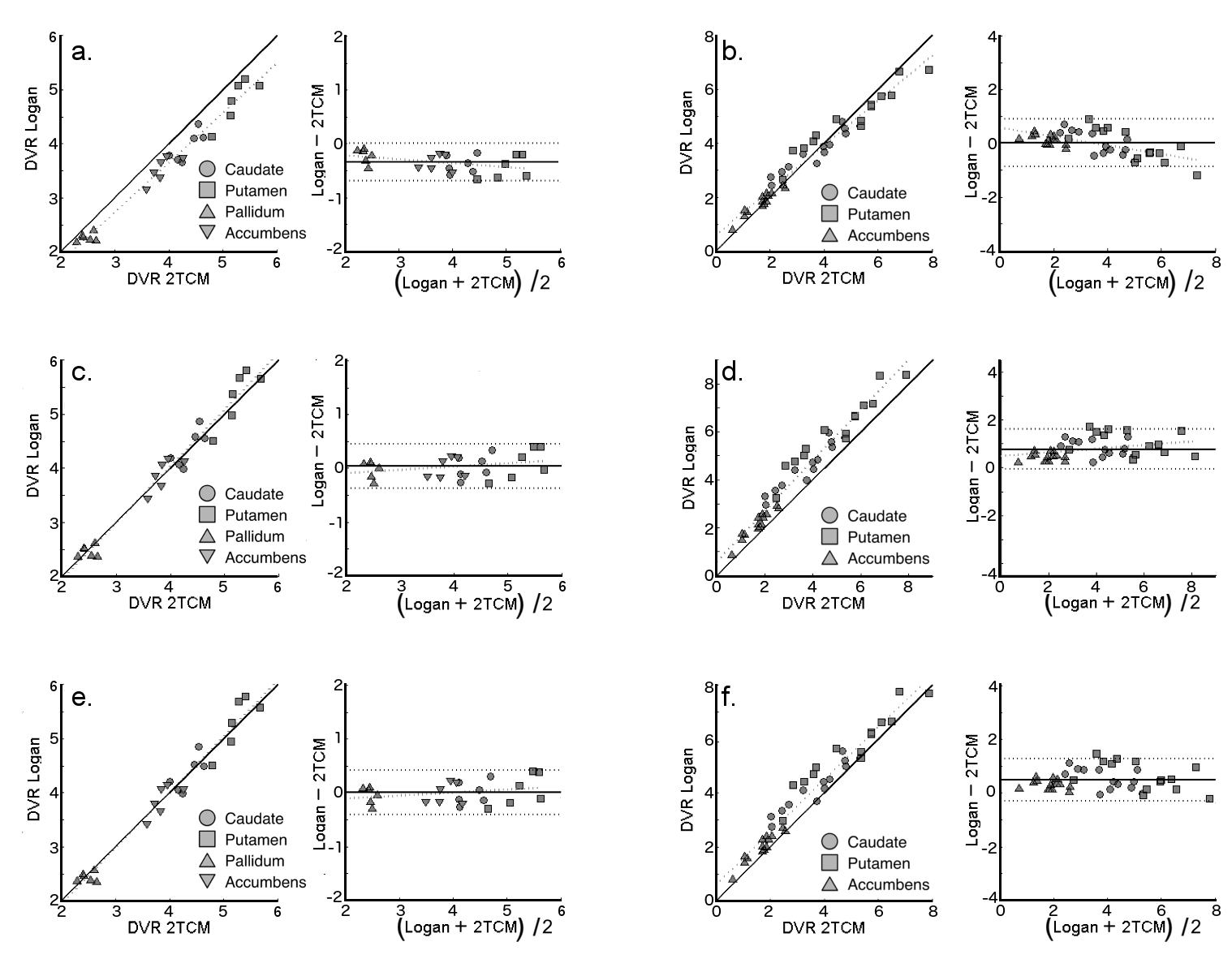


**Figure S1** Distribution volumes ratios (DVR) obtained with 2TCM plotted against those obtained with Logan ref, and corresponding Bland-Altman plots. DVR from Logan ref is: not corrected (**a,b**), corrected using measured values for *α* (**c,d**), and calculated values for *α* (**e,f**). Left column (**a,c,e**) shows [^11^C] raclopride data, and right column (**b,d,f**) shows [^18^F] MNI-659 data. Corrections are performed without assuming that the signal contribution from blood volume in the target region is negligible.

**S2. Including Pallidum in the analysis of [^18^F]MNI-659 data**

For [^18^F]MNI-659 data, the results for Pallidum differed from the rest of the regions included in the analysis. This might be related to the inclusion of both the internal and external structure, as well as a lower delivery of radioligand. Since the evaluation of the new method was performed across ROIs, the analysis was conducted both with and without Pallidum included in the ROI set. The figure corresponding to figure 5 in the manuscript (and figure S1 in the supplementary material) with Pallidum included is shown below.


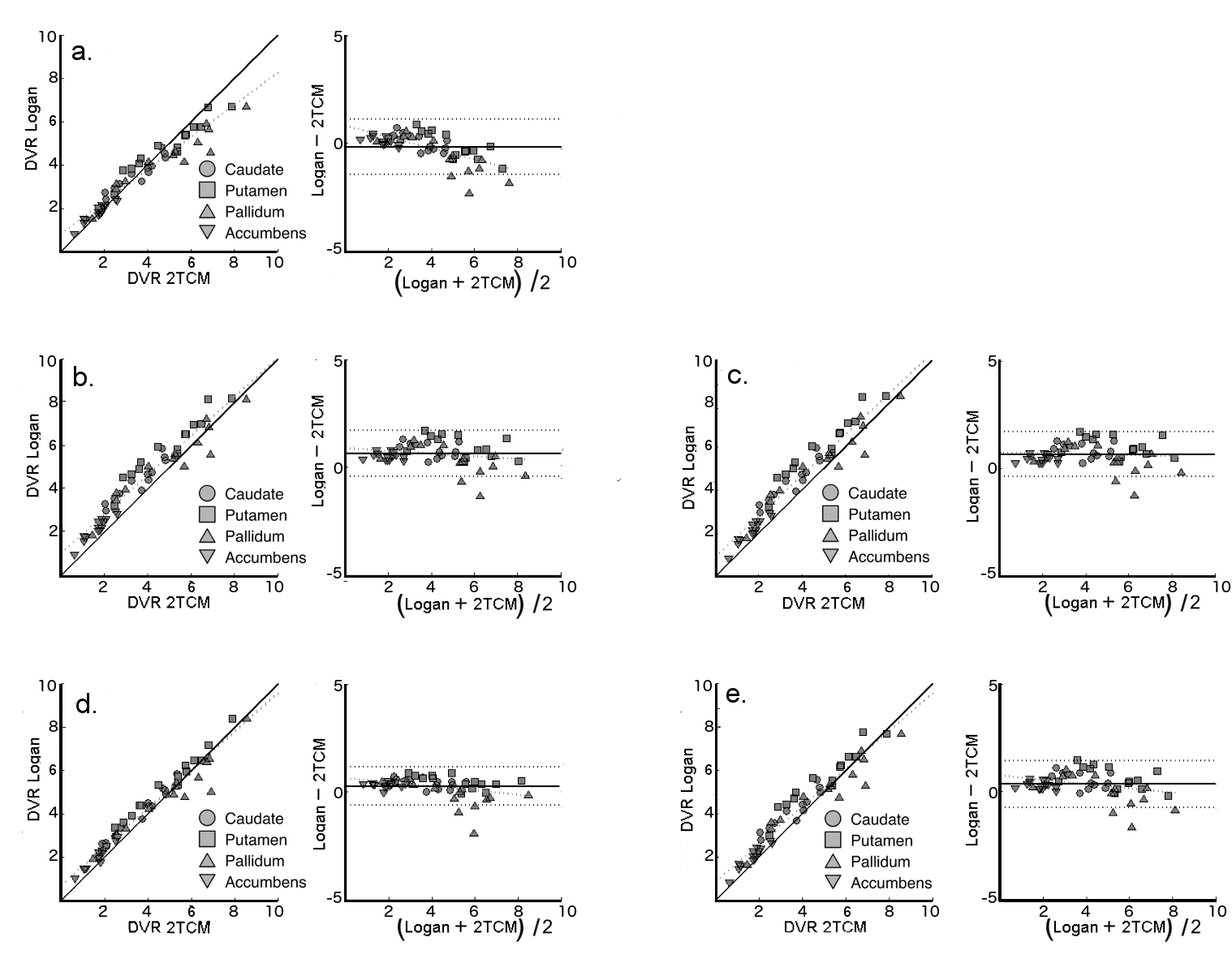


**Figure S2** Distribution volumes ratios (DVR) obtained with 2TCM plotted against those obtained with Logan ref, and corresponding Bland-Altman plots for [^18^F]MNI-659 data. DVR from Logan ref is: not corrected (**a**), corrected using measured values for *α* (**b,c**), and calculated values for *α* (**d,e**). For the plots shown in **b** and **d**, the correction was performed assuming negligible contribution from blood in the target region (i.e., equation (11) in the manuscript, and right column (**c,e**) shows correction assuming blood signal contribution in both target and reference region (i.e., equation (S4) in supplementary material).
